# Takotsubo cardiomyopathy vs acute myocardial infarction: diagnostic utility of subtle ECG differences

**DOI:** 10.1186/1865-1380-4-17

**Published:** 2011-04-21

**Authors:** Asma Saba Syed, Umair Khalid

**Affiliations:** 1 Medical College, Aga Khan University Stadium Road, PO Box 3500, Karachi 74800, Pakistan

## Abstract

The clinical findings of Takatsubo Cardiomyopathy and acute myocardial infarction can be very similar. While Takatsubo cardiomyopathy rarely leads to severe complications, acute myocardial infarction can be life threatening. Treatment of both these conditions is different and so it is imperative for clinicians to have a high index of suspicion for either. Several EKG differences between the two entities have been proposed. This article summarizes the EKG changes most likely seen in Takatsubo cardiomyopathy and compares them to those seen in Acute Myocardial infarction.

## 

Takotsubo cardiomyopathy (TCM) is a rare clinical entity, having clinical and electrocardiographic (ECG) findings very similar to those found in acute myocardial infarction (AMI). First described in a group of postmenopausal Japanese women, it derives its name from the striking resemblance of its typical ventriculogram findings to the Japanese takotsubo octopus trap. Its prevalence ranges from 0.7% to 2.5%, primarily affecting the elderly female population bracket [1,2]. Although the pathogenesis is still a matter of on-going debate, catecholamine-mediated cardiotoxicity provoked by emotional or physical stress has been proposed as an explanation [1].

Clinical distinction between the TCM and AMI is of paramount importance since the latter is a medical emergency. On the other hand, the prognosis of takotsubo cardiomyopathy is generally considered favorable. Although QT interval prolongation in TCM carries a risk for arrhythmias in the acute and sub-acute period, sudden cardiac death and life-threatening ventricular arrhythmias are uncommon [1,3].

Recent studies indicate that certain ECG findings can assist in this diagnostic predicament. In TCM, the most common ECG finding on presentation is ST segment elevation (STE) in the precordial leads. However, the extent of this STE is less than that found in AMI, and there is no concurrent ST segment depression seen in other leads. A retrospective case series demonstrated that higher ST elevation voltage in leads V4-V6 than V1-V3, and absence of pathologic Q waves and reciprocal changes in the inferior leads showed high sensitivity and specificity to help differentiate TCM from AMI [4]. Furthermore, prominent T waves also emerge and gradually deepen to their first negative peak within 3 days. These T waves then turn shallow, before becoming significantly deeper on their second negative peak in 2-3 weeks [1]. Lastly, the presence of prominent U waves has been proposed as an additional ECG finding to support the diagnosis of TCM [1].

Clinicians should have a high index of suspicion for TCM among elderly women who present with angina following a history of emotional or physical stress. As mentioned, TCM patients generally have lower ST elevation voltage and less frequent reciprocal changes compared to patients with AMI. Although these ECG findings add incremental diagnostic value, the definitive diagnosis still requires invasive cardiac testing [4]. Knowledge of these ECG differences can guide clinicians in distinguishing between TCM and AMI in clinical settings so that the appropriate management steps can be carried out.

## Competing interests

The authors declare that they have no competing interests.

## Authors' contributions

UK performed the literature search, interpreted the data and drafted the manuscript. ASS conceived the idea, performed the literature search, interpreted the data, drafted the manuscript and critically revised it. All authors have read and approved the final manuscript.
